# Evidence‐based complementary feeding recipe book for Kenyan caregivers: A novel approach

**DOI:** 10.1111/mcn.13475

**Published:** 2023-10-03

**Authors:** Alyssa Lowe, Amy Callis, Ann DiGirolamo, Amy W. Girard, Amma Boakye, Emily Ogutu, Esther Omosa, Frida Okeyo, Lawrence Odollo, Betty Samburu, Caroline Arimi, Penjani Kamudoni, Wendy Gonzalez, Patrick Codjia, Laura Kiige

**Affiliations:** ^1^ Georgia Health Policy Center Georgia State University Atlanta Georgia USA; ^2^ Devi Partners SAN CARLOS California USA; ^3^ Hubert Department of Global Health, Rollins School of Public Health Emory University Atlanta Georgia USA; ^4^ International Livestock Research Institute Nairobi Kenya; ^5^ Department of Community Health and Development, School of Public Health Great Lakes University of Kisumu Nairobi Kenya; ^6^ United Nations Children's Fund Kenya Country Office Nairobi Kenya; ^7^ Kenya Ministry of Health Nairobi Kenya; ^8^ Global Alliance for Improved Nutrition Geneva Switzerland

**Keywords:** behaviour change, complementary feeding, Kenya, visual communication

## Abstract

The Kenyan Ministry of Health (MOH) and a consortium of nutritionists, researchers and communication, and design specialists developed a novel approach to create an evidence‐based recipe book promoting complementary feeding (CF) in Kenya. The ADAPT approach includes five steps: applied research (A), dialogue with stakeholders (D), adapted behaviour change communication (BCC) theories (A), purpose‐driven visual communication (P), and tailoring to priority audiences (T). Through this approach, the recipe book addresses key knowledge gaps using behaviour change theories and visual communication best practice to increase accessibility, acceptability, retention and motivation for behaviour change. The book addresses barriers to CF identified through formative applied research. Dialogue with stakeholders helped ensure cultural appropriateness and the book's alignment with MOH recommendations and key messages. The book uses behaviour change theories to approach the reader in a respectful way that motivates behaviour change. The use of consistent, purpose‐driven visuals helps ensure key messages are easily understood and accessible to all caregivers regardless of literacy level. The book's tone and content are tailored to its audiences’ attributes, needs and preferences. This five‐step process inspired the development of ADAPT, a novel approach that integrates behaviour change and visual communication for greater impact. This paper outlines how the consortium used the ADAPT approach to develop an evidence‐based book that thoughtfully and holistically addresses a wide range of barriers, provides practical solutions and increases self‐efficacy around CF. It offers a blueprint for public health practitioners from any field who are interested in using visual behaviour change communication to promote healthy behaviour.

## Key messages

- A complementary feeding recipe book was developed for Kenyan caregivers.
- Development of the book included an evidence‐based five‐step approach, ADAPT, to increase accessibility, acceptability, retention and motivation for behaviour change.
- ADAPT steps include (1) applied research, (2) dialogue with stakeholders, (3) adapted BCC theories, (4) purpose‐driven visual communication and (5) tailoring to the audience.
- The steps supported the development of an evidence‐based book that thoughtfully and holistically addresses barriers, provides practical solutions and increases self‐efficacy.
- The novelty of this approach lies in its use of behaviour change theory and visual communication to reinforce key messages and change behaviour.

## Graphical abstract

A complementary feeding recipe book was developed for Kenyan caregivers. Development of the book included an evidence‐based five‐step approach, ADAPT: (1) applied research, (2) dialogue with stakeholders, (3) adapted BCC theories, (4) purpose‐driven visual communication, and (5) tailoring to the audience. The steps supported the development of an evidence‐based book that uses behaviour change theory and visual communication to thoughtfully and holistically address barriers, offer practical solutions, and increase self‐efficacy.

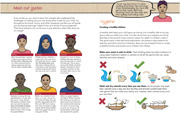

## 1 INTRODUCTION

Kenya has made strong progress in improving infant and young child nutrition. Between 2008 and 2014, the proportion of stunted children decreased from 38% to 26%, and the proportion of children under 5 who were underweight decreased from 16% to 11% (Kenya National Bureau of Statistics, 2015). Nearly half of the children 6–23 months old are offered the minimum recommended number of meals each day for their age, and 41% were offered the minimum dietary diversity of four or more food groups per day. However, 77% of children under 2 still do not consume a minimum acceptable diet (minimum daily meal frequency for age and adequate diversity of four or more food groups each day) (Kenya National Bureau of Statistics, 2015). Based on early successes and the desire for continued improvements, the Ministry of Health (MOH) is focusing on promoting complementary feeding (CF) best practices to improve the percentage of children under 2 whose diets meet the minimum acceptable standards.

Appropriate CF from 6 months to 2 years of age is critical for optimal health. Adequate nutrition during early childhood is necessary for healthy growth and development. The consequences of poor nutrition include mortality, morbidity and delayed mental and motor development in childhood; and are associated with impaired intellectual performance, work capacity, reproductive outcomes and overall health throughout adulthood.

To promote optimal CF, MOH set CF‐related goals in the National Nutrition Action Plan, the National Strategy for Maternal Infant and Young Child Nutrition (MIYCN), and the MIYCN National Operational Guidelines (Kenya Ministry of Health, 2012, 2013, 2021). In 2018, MOH developed a National Guide to CF to ‘improve communication to caregivers of children six to twenty‐three months…as well as provide recommendations on how to develop and roll‐out CF recipes using locally available foods’ (Ahoya et al., 2019; Kenya Ministry of Health, 2018).

To further these efforts, UNICEF partnered with the Bill and Melinda Gates Foundation on the Regional Initiatives for Sustained Improvements in Nutrition and Growth (RISING), which aims to strengthen the organisational and technical leadership of regional platforms to support key evidence‐based interventions to improve maternal and child nutrition. As an addendum to the RISING project the UNICEF Kenya Country Office (KCO), Global Alliance of Improved Nutrition (GAIN) and MOH, commissioned a consortium to develop a recipe book of complementary foods for infants and young children in Kenya.

Applied research seeks to provide innovative solutions to issues. Given that the project's scope focused on the practical development of an innovative product, the consortium purposefully used applied research to support the project rather than using traditional academic research to complement the existing knowledge base. Formative applied research included a scoping desk review that found an abundance of existing research on CF behaviours in Kenya; however, the review did not find any literature that explored the relationship between CF, behaviour change theory and visual communication or strategies for developing visual behaviour change tools for CF. Thus, the consortium designed a solution‐driven process that facilitated the development of ADAPT, a novel approach integrating applied research and stakeholder engagement with multiple behaviour change theories and the strategic use of visual communication research. Unlike other approaches, ADAPT combines evidence‐based BCC theories and visual communication best practices to foster acceptance, efficacy and adoption of CF practices among Kenyan caregivers.

The ADAPT approach includes five steps: applied research (A), dialogue with stakeholders (D), adapted behaviour change theories (A), purpose‐driven visual communication (P) and tailoring to the priority audience (T). This paper describes the ADAPT approach and how the consortium used it to develop the recipe book. It explores opportunities for evaluation and potential applications to other public health efforts.

## 2 METHODS

**Figure 1:**
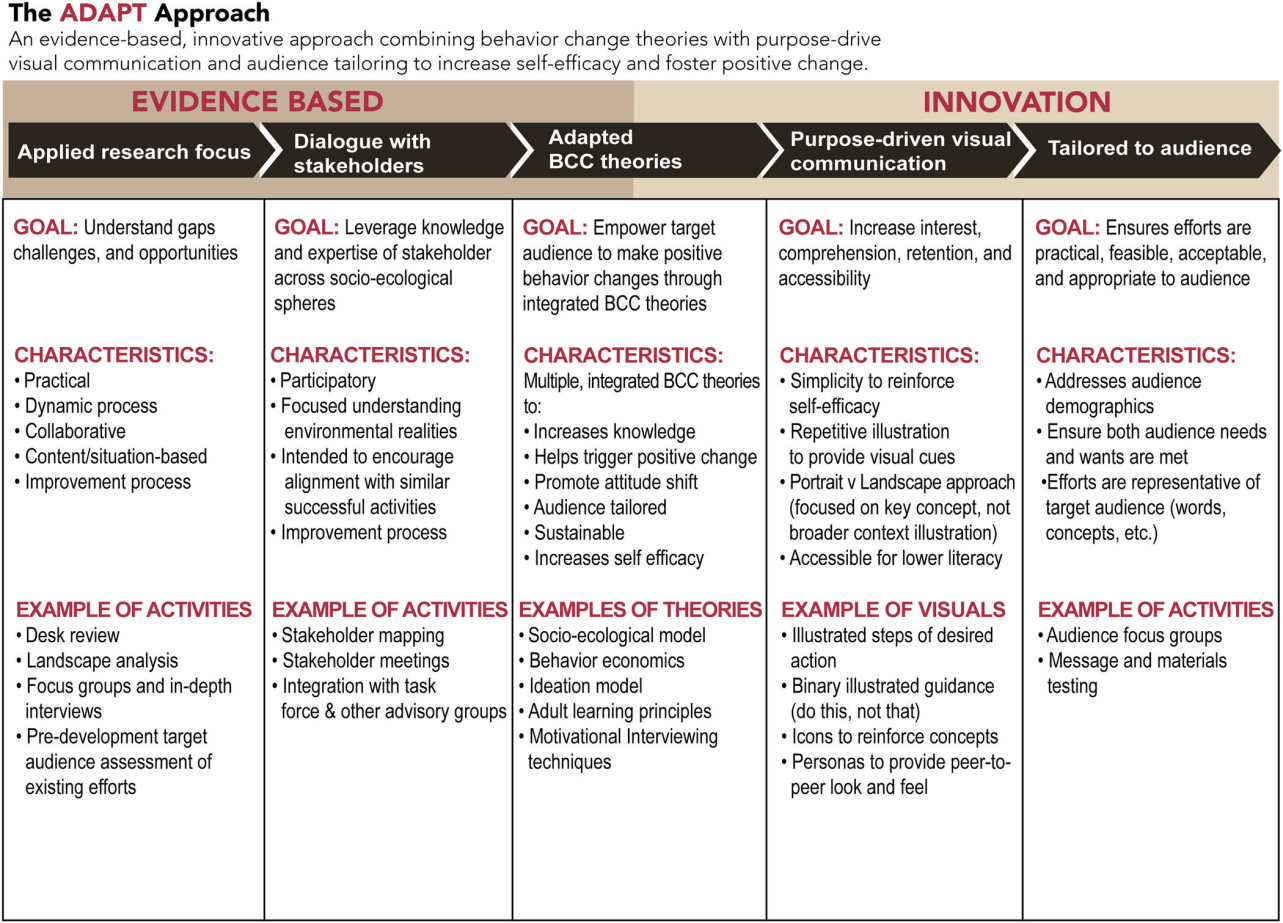
ADAPT approach framework.

Figure 1 outlines the goals, characteristics and activities associated with each of ADAPT's methodological steps.

### 2.1 Applied research

Formative applied research ensured the book employed practical solutions based on contextual factors. Formative research included (1) a scoping desk review of existing literature, (2) a landscape analysis of Kenyan CF cookbooks and cookbooks using visual communication and (3) primary data collection. The Internal Review Boards of Georgia State University (GSU), Emory University, and Great Lakes University of Kisumu (GLUK) approved protocols. Additionally, the International Livestock Research Institute (ILRI) received approval from the Kenya National Commission for Science, Technology and Innovation (NACOSTI).

#### 2.1.1 Scoping desk review

The purpose of the scoping desk review was to provide insight into the Kenyan CF landscape, generate recommendations for the recipe book's design and content, and shape the purpose and scope of primary data collection. The consortium prioritized sources related to the RISING project (project documents and reports) and those authored by MOH, GAIN and UNICEF. Additional sources included peer‐reviewed literature, World Health Organization guidelines, CF behaviour change communication materials, curricula, reports and websites from INGOs, NGOs and Community‐Based Organizations (CBOs) and large‐scale household surveys.

Over 40 search terms were used including topics related to infant and young child feeding (IYCF), the recipe book's target audiences, location (Kenya, East Africa), food culture and food systems.

#### 2.1.2 Landscape analysis

The purpose of the landscape analysis was to explore (1) existing CF recipe books available to Kenyan caregivers, (2) publications outlining the development and evaluation of those books and (3) explore visual communication strategies used in cookbooks generally. Landscape analysis sources included peer‐reviewed literature, 18 Kenyan cookbooks and recipe cards, 24 illustrated cookbooks and visual communication texts. Search terms related to the landscape analysis included more than 24 terms related to illustrated cookbooks, Kenyan cookbooks and visual communication.

#### 2.1.3 Primary data collection

The consortium conducted primary data collection to inform the design and content of the recipe book by (1) increasing understanding of current CF practices and how optimal CF practices are promoted; (2) exploring strategies for using a recipe book to influence CF behaviour; and (3) gathering information on factors critical to developing nutrient dense, accessible recipes.

The Kenya‐based research team purposively selected subcounties with input from stakeholders at MOH, GAIN and UNICEF to ensure the social, economic, ethnic and cultural diversity of the country was represented. Forty‐three subcounties in 16 counties were chosen based on food culture, food availability and location of shared markets. Locations included central and border locations and an urban setting with a diverse population (Nairobi).

Research teams led by GLUK and ILRI conducted 92 in‐depth interviews (IDIs) (five per county) with participants from two priority populations that represented the recipe book's target audiences: (1) caregivers including mothers, fathers, and grandparents (*n* = 45); and (2) those who educate and train caregivers on IYCF best practice including nutrition officers, front‐line health workers and Community Health Volunteers (CHVs) (*n* = 47). The sample size was determined a priori and based on expected reasonable coverage (Patton, 1990).

Respondents were between the ages of 18 and 41 and the sample was 65% female. The research team recruited Health Workers and CHVs from Nutrition Technical Fora, County Steering Groups, County Executive Health Officers, and Sub‐County Public Health Officers. Caregivers were identified using maps developed by Sub‐County Public Health Officers and CHVs and recruited via phone using contact lists. Data collectors used purposive snowball sampling to obtain a diverse mix of age groups and cultural backgrounds among participants by asking interviewees to recommend others within their community who could provide a unique perspective.

Due to COVID restrictions, focus group discussions and community‐based participatory methods were not possible. Data collectors conducted interviews via phone and obtained informed consent verbally at the beginning of interviews. The consortium developed 14 open‐ended interview questions and corresponding probes to guide the discussions.

#### 2.1.4 Data analysis

The research team managed and analyzed desk review and primary data sources in NVivo and MAXQDA using a structured codebook. The team developed detailed definitions for each code to ensure clarity and consistency of use. Members of the consortium from GHPC and Emory University coded the data, with multiple analysts coding each interview. Data analysts from GHPC and Emory compared coded interviews for intercoder reliability and addressed discrepancies. Data analysts facilitated sense‐making sessions with in‐country data collection teams led by GLUK and ILRI and presented preliminary findings to the recipe book consortium who supported verification and interpretation of the findings.

### 2.2 Dialogue with stakeholders

The consortium identified stakeholders across socioecological spheres of influence and key sectors with guidance from UNICEF, GAIN and MOH (Kilanowski, 2017). This mapping exercise ensured broad participation across socioecological spheres of influence and sectors and determined how and when stakeholders should be involved in the project.

MOH‐led technical working groups (TWGs) including the Nutritional Information and the Maternal Infant and young Child Nutrition (MIYCN) TWGs reviewed research protocols. A task force of nutrition experts from academia, MOH, UNICEF, and other international nongovernmental organisations (INGOs) was assembled to review recipe book content and design. The TWGs ensured appropriateness, technical accuracy, and alignment with MOH guidance. MOH identified county and sub‐county stakeholders to support community entry during data collection and community‐level stakeholders participated in formative research. The consortium also created a Facebook page to gather informal input from a wider array of stakeholders.

### 2.3 Adapted BCC theories

The novelty in the approach to the recipe book's development lay in the integration of BCC theories and visual communication research. Members of the consortium with BCC expertise identified BCC theories specific to addressing gaps and opportunities including:

- Ideation model: The model explains how new ideas and behaviours are spread by communication and social interaction. The model supposes that behaviour is influenced by multiple social and psychological factors, as well as skills and environmental conditions (Health Communication Capacity Collaborative, 2015).
- Adult learning theory: A conceptual framework for adult learning focused on education through praxis, allowing learners to reflect on their world, thus fostering self‐efficacy to change it (Dirkx, 1998).
- Motivational interviewing (MI): A collaborative communication style framed in acceptance, comprehension and compassion to strengthen internal motivations for new behaviours (Szczekala et al., 2018). The approach fosters self‐efficacy and a desire to engage in behaviour change (Rollnick & Miller, 1995).
- Nudge theory: A behaviour economics theory that provides supportive framing of issues and positive reinforcement as well as indirect suggestions and small clues to influence behaviour and facilitate positive decision‐making (Harrison, 2020).
- Socioecological model: A public health framework that acknowledges the interplay between different spheres of influence that impact decision‐making (Kilanowski, 2017).

The consortium also leveraged a conceptual framework traditionally used to increase participation in clinical trials that includes the concepts of appropriateness, acceptability, availability and accessibility (Fern et al., 2014) to ensure the book's appeal and utility.

### 2.4 Purpose‐driven visual communication

Visual communication improves comprehension, and retention of important public health concepts designed to improve individual and community health. The strategic, evidenced‐based use of visual communication ensured a recipe book that is applicable and accessible to all Kenyan caregivers by incorporating evidence‐based visual communication best practices including:

- Pictography and iconography: Utilization of culturally appropriate images as part of public health interventions can improve effectiveness in lower literacy populations (Paige et al., 2017; Pater, 2016). This is a useful strategy given that low health literacy (and low/lower general literacy) can induce anxiety in learning new behaviours, impeding comprehension and self‐efficacy (Moss, 2019; Wolpin et al., 2016).
- Visual complexity and concept illustration: Limiting the number of visual elements makes messages easier to understand (Heath & Heath, 2007). Simple graphics with limited visual complexity ‘emphasize important information and de‐emphasizes irrelevant details’ and help users quickly understand and retain concepts and instructions (Agrawala et al., 2011). Simple images that are relevant to the key message are also more highly valued by readers than detailed/decorative illustrations (Tufte, 2006).
- Colour Palette: Strategic use of colour can clarify messages, provide visual cues and engage audiences (Park & Tang, 2019). Evidence suggests that colour can affect cognitive understanding, task performance and task accuracy (Kwallek et al., 2007). Colour palettes also have strong cultural preferences (Park & Tang, 2019).
- Positive visuals to reinforce emotions: There is an established link between emotion, design, and cognition. Positive and pleasing imagery can evoke positive emotions that enable learning (Moss, 2019; Nadler et al., 2010). Positive emotions are also linked to engagement and motivation as well as improving retention of key concepts (Isen, 2001). Content that is both engaging and encouraging can make an experience more positive, memorable, and more likely to be repeated (Moss, 2019).
- Representation through imagery: Visual representation can be a powerful tool in behaviour change but can also unintentionally reinforce stereotypes and negative norms and perceptions (Wilson, 2011). For visual representation to be most effective as part of a behaviour change strategy, it should be purposeful in representing empowered individuals with positive insights to their own lived experiences (Lindsey, 2013). The influence of empowered visual representation can affect perceptions across the socioecological model, supporting broader social norming efforts (Allagui & Al‐Najjar, 2018). This socioecological effect is important given that an individual's agency can be dependent on factors outside their control (Galiè & Farnworth, 2019).

### 2.5 Tailored to audience

Tailored informational interventions (TI) or customising health information to meet specific demographic and/or individual characteristics have a greater impact on health behaviour change and produce better health outcomes than standard non‐TI (Ryan & Lauver, 2002). There is also some evidence to suggest that tailored messages are more affective when paired with visuals (Noar et al., 2007).

### 2.6 Ethics  statement

The Internal Review Boards of Georgia State University (GSU), Emory University, and Great Lakes University of Kisumu (GLUK) approved protocols. Additionally, the International Livestock Research Institute (ILRI) received approval from the Kenya National Commission for Science, Technology, and Innovation (NACOSTI).

## 3 RESULTS

### 3.1 Step 1: Applied research

Findings from the scoping desk review, and IDIs uncovered four key themes: (1) nutritional practices and dietary diversity, (2) feeding practices, (3) cooking practices and (4) social norms. Major findings included:

- Economic and seasonal availability impact dietary diversity.
- Cultural norms and perceptions negatively impact CF.
- There is a lack of awareness around feeding frequency and quantity.
- CF is introduced prematurely.
- There is a lack of awareness around safe cooking practices, food safety and hygiene.
- There is a wide variety of food preparation and cooking equipment.
- Gender and family roles impact food production, purchases, and preparation.
- Target audience perception of cookbooks was not positive. Respondents felt the cookbooks typically focus on *how* to cook, something they have high confidence in, rather than *what* to cook, which had more utility for them.

Table 1 summarizes key findings by theme and provides an overview of how the findings informed the recipe book's content and design.

**Table 1:** Summary of key formative research findings, associated nutritional goals and the book's content and design strategies by theme.

Summary of key research findings	The recipe book's nutrition‐related goals	Content and design strategies
*Nutritional practices*		
*Dietary diversity* Poor dietary diversity was the most frequently mentioned challenge in the literature.	Provide caregivers with knowledge and strategies for increasing children's dietary diversity	In alignment with MOH guidance, the book includes recommendations to give food from four food groups per day and from all age‐appropriate food groups per week. Dietary diversity is explained and clearly illustrated using evidence‐based visual tools including a food wheel. The food wheel helps readers visualize food groups and offers potential ingredient substitutions. Weekly meal plan examples are provided for each age group to help caregivers plan for appropriate dietary diversity. Recipes include the number of food groups included in the recipe, and examples of additions from other food groups to increase dietary diversity.
*Affordability and availability* Affordability and availability were considered the primary barriers to dietary diversity. Ensuring good hygiene, not overcooking foods, and increasing micronutrient bioavailability help caregivers ensure their children are getting the highest nutritional return on the family's investment. Seasonal and regional availability, and drought‐related food scarcity influence availability.	Provide caregivers with recipes that include affordable ingredients that are available across Kenya throughout the year.	Recipes include nutrient dense and affordable ingredients identified by the CONGA and affordability studies. Icons are used to identify these ingredients. Recipes include food processing and preparation tips to ensure caregivers are getting the highest nutrient value for their money. For example, the book includes instructions on how to (1) ferment grain to increase its nutrient value (2) soak grains to make their micronutrients easier to absorb and (3) steam—as opposed to boil—vegetables to help ensure nutrients are not lost during cooking. Recipes include interchangeable ingredients that provide nutritionally equivalent substitutes, allowing caregivers to select ingredients based on local availability and affordability.
*Cultural barriers to dietary diversity* Caregivers categorise foods as problematic if they were perceived to be a choking hazard, difficult to digest, or lack nutritional benefits (Pelto & Armar‐Klemesu, 2015). In depth interview participants noted that eggs make children ‘unable to talk,’ animal organs including liver (an affordable and nutrient‐dense food) were thought to make children develop a ‘heavy tongue,’ and red meat was thought to ‘make the child weak.’ However, these foods are both nutrient dense and safe when prepared properly (Kram et al., 2016).	Address potentially harmful taboos and provide caregivers with strategies for making healthy meals based on cultural preference.	Personas are used to acknowledge concerns and provide peer‐to‐peer style engagement to accurately address misinformation. Recipes explain preparation techniques and food modifications to make foods safe for children. Interchangeable ingredients allow caregivers to make substitutions based on cultural preference.
*Feeding practices*		
*Lack of awareness around feeding frequency and quantity* Based on the 2018 DHS, 51% of children 6–23 months were offered the minimum recommended number of meals each day for their age and 22% met criteria for a ‘minimum acceptable diet’ (Kenya National Bureau of Statistics, 2015). In interviews, caregivers were uncertain how often and how much children of different ages should be fed. When asked about feeding frequency, caregivers provided inconsistent answers, with the number of meals ranging from 3 to 7 a day within the same age group.	Provide caregivers with guidance on age‐specific feeding frequency and quantity.	The recipe book includes guidance on quantity and frequency of feeding for each age group throughout and includes age‐specific weekly meal plans. Additionally, it reinforces the need to continue breastfeeding as part of CF. Infographic visuals were developed to provide easy‐to‐understand feeding schedules.
*Premature CF (before 6 months)* CF initiation in Kenya may begin as early as 2 months (Kimani‐Murage et al., 2011; Matsuyama, 2013). Factors that influence CF initiation include household finances and cultural norms (Matsuyama, 2013). Problems with breastfeeding were the most mentioned reason for premature CF.	Provide caregivers with information about exclusive breastfeeding and appropriate initiation of complementary feeding.	The book includes information on when to initiate complementary feeding and addresses concerns that lead caregivers to initiate complementary feeding early including a section on breastfeeding. It clearly explains the health benefits to exclusive breastfeeding for the first 6 months and continued breastfeeding until 24 months.
*Cooking practices*		
*Sources of information on food preparation* Caregivers shared they receive infant and young child nutrition (IYCN) messages from many sources including CHVs; however, information from friends, family and neighbours was most influential.	Acknowledge caregivers' existing skill and capacity. Use nontechnical terms, simple instruction and familiar tone.	Personas in the book deliver key messages ‘to caregivers from caregivers'.
*Perceptions and use of cookbooks* While there was interest in learning what to cook to ensure a healthy diet, cookbooks are perceived to be for people who do not know how to cook, and caregivers—specifically mothers—already know how to cook. Interview participants who had used recipe books before cited complexity and time as barriers to use. Health workers cautioned against making the book too long or the recipes too complex and unfamiliar.		Personas in the book acknowledge caregivers' existing knowledge, skill and capacity. Recipes are familiar. Instruction focuses on how to adapt and supplement family favorites to meet IYC's nutritional needs. The book includes an introductory education section in addition to recipes. Recipes are developed to be limited in steps to be viewed as simple and not time intensive.
*Measurement* Caregivers rarely included conventional measurements when explaining how to prepare an example dish. When probed about measurement, responses included terms like ‘a handful of flour'; references to estimated size such as ‘palm‐sized'; ratios; and count such as ‘four–six Irish potatoes'.	Ensure recipes can be used effectively with a range of available cooking equipment.	Recipes use ratios and hand‐size comparisons in addition to standard measurements.
*Cooking Time* Foods are cooked over different heat sources using different fuels, so cooking times vary depending on tools and fuel used. Interviewees determined cooked food's ‘doneness' by taste, look (‘when it bubbles'), smell, temperature and texture (‘when it is easy to mash').		Recipes acknowledge different heating sources and fuels and use colour, texture and consistency as indicators of ‘doneness'.
*Food Safety and Hygiene* Desk review findings illustrated the link between nutrition, water, sanitation and hygiene (WASH). Caregivers shared meal preparation techniques that could have negative health impacts depending on storage and reheating practices. Health workers recommended that hygiene messages be included in the recipe book.	Provide caregivers with key WASH messages relevant to IYCN.	The recipe book includes WASH messages throughout. Some food hygiene practices have the potential to reduce foods' nutrient value. For example, while caregivers should cook food completely to ensure it is safe to eat, they must also avoid overcooking to ensure nutrient density is not lost. Recipes provide preparation tips to help caregivers find the right balance; for example, cooking hard root vegetables (orange‐fleshed sweet potatoes, carrots, etc.) first before adding the softer vegetables (tomatoes, greens) to avoid overcooking.
*Social norms*		
Gender and family roles are the predominant social norms impacting food production, purchasing, preparation and provision (Harvey et al., 2017; Kram et al., 2016). Interview participants across target groups and regions shared that the father's role is to purchase food, provide mothers with money to purchase food and produce food (farming and animal husbandry). Grandmothers and other elders educate parents on what and when to feed young children. Given their influence, some health workers identified grandmothers as a potential barrier to behaviour change.	Reflect existing social norms and encourage strong familial support from fathers and grandmothers.	Personas include a father and a grandmother to deliver messages based on both their existing and potential roles in IYCF.

Abbreviations: CF, complementary feeding; DHS, demographic health survey; IYCF, infant and young child feeding; MOH, Kenyan Ministry of Health.

According to IDIs, caregivers were not interested in learning new, complex recipes. Thus, rather than introduce new recipes, the book focused on adapting familiar recipes. This decision also aligned with behaviour change strategies that indicate small changes are easier to adopt than larger ones (Damschroder et al., 2010). The consortium took great care when writing these adapted recipes to ensure they were as simple as possible with limited steps.

Landscape analysis found that many existing CF recipes books and cards include technical information not relevant or appropriate to the intended target audience. For example, recipes used academic jargon, used inconsistent measurements and had varying levels of complexity. Existing sources did not consistently include guidance on age‐appropriate quantities and consistencies or strategies to increase dietary diversity.

The team worked to ensure the content was tailored to the priority population by avoiding technical language and ensuring consistency and clarity across all recipes. The consortium organized recipes by age group, included age‐based serving sizes and thoroughly explained age‐appropriate quantities and consistencies.

Images in existing recipe books were most often photographs of foods and did not provide opportunity for additional learning such as appropriate food hygiene and safe cooking practices. Nutrition cards were most frequently illustrated in a ‘story telling’ approach that provided significant extraneous detail not relevant to the demonstrated behaviours. Illustrated recipe books focused on appeal rather than understanding, with photographs and illustrations that were not visual communication focused. Thus, the team prioritized visual communication for the purpose of behaviour change as described throughout this section.

### 3.2 Step 2: Dialogue with stakeholders

Expert review ensured that recipes met age‐specific nutrient needs, were responsive to critical micronutrient gaps, appropriately promoted dietary diversity and were aligned with MOH guidelines and key messages. MIYCN experts from MOH requested that the book's nutrition education section align with the ‘FATVAH’ framework, which includes frequency, amount, texture, variety, active feeding, and hygiene (Figure 3) (Ahoya et al., 2019). Alignment with MIYCN training resources will allow health workers and volunteers to incorporate the recipe book into their existing training activities and help ensure caregivers are seeing consistent messages in all MOH materials. Experts also provided guidance on how to address practical issues like measurement and cooking times by providing recommended language that would be clear to caregivers. For example, determining ‘doneness’ by taste, look (‘when it bubbles’), smell, temperature, and texture (‘when it is easy to mash’).

Partners at GLUK and ILRI collected feedback on the book's icons and graphics from field staff and caregivers. These community‐based stakeholders suggested that illustrations of caregivers look less polished to make them more relatable. Stakeholders also provided input on food graphics to ensure they were easily recognizable. For example, stakeholders suggested replacing a square milk carton with the more common triangular carton.

### 3.3 Step 3: Adapted BCC theories

**Figure 2:**
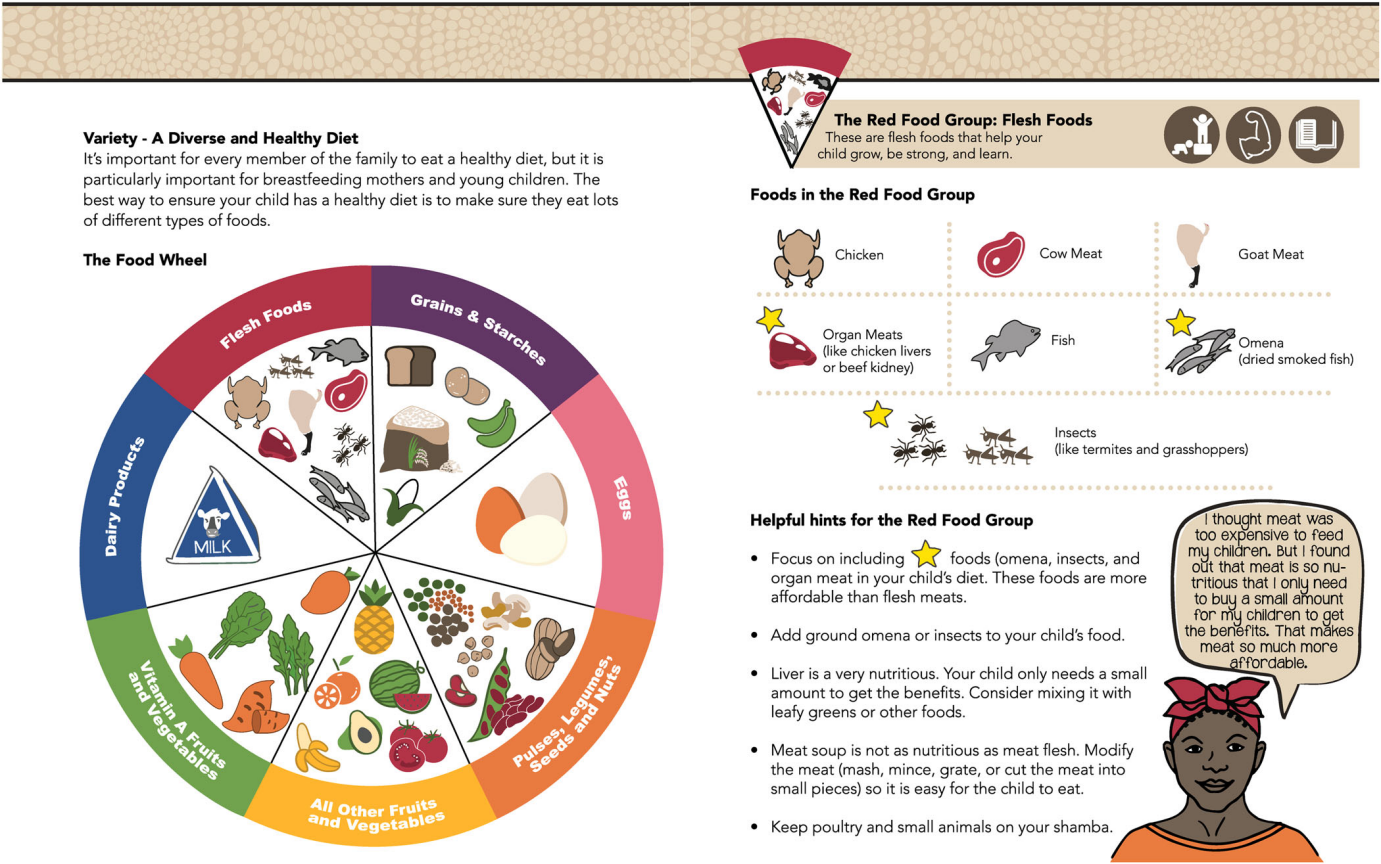
Recipe book food wheel.

BCC and adult learning theories were used to empower and improve caregivers’ self‐efficacy. Each key element in the book leveraged BCC theories depending on the barrier they were addressing. For example, the consortium used a food wheel to promote dietary diversity (Figure 2). The wheel used adult learning theory by providing clear strategies to increase dietary diversity that could be implemented immediately and used the ideation model to ensure the wheel focused on building knowledge.

**Figure 3:**
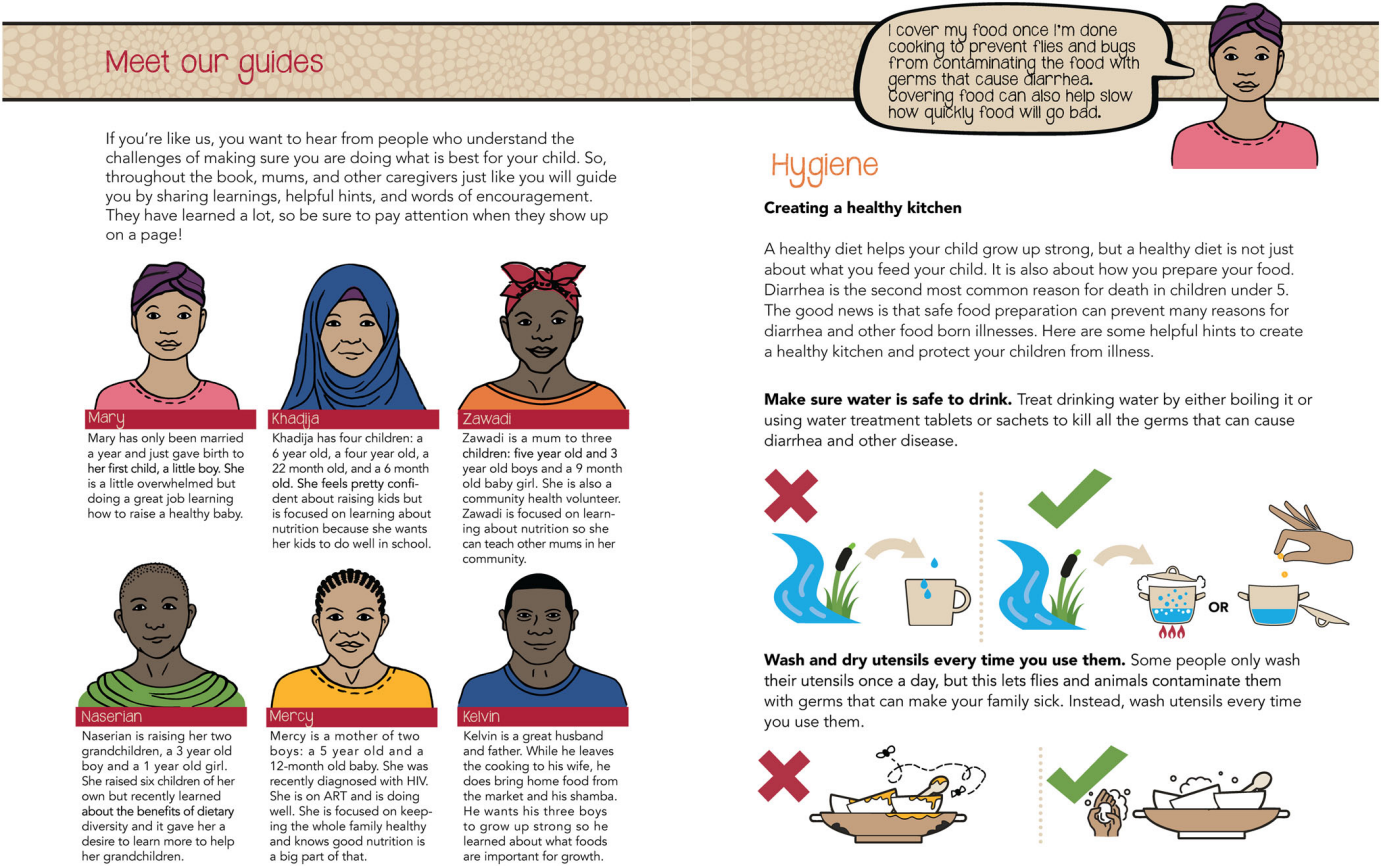
Personas and FATVAH excerpt.

MI techniques ensured the book used accepting and compassionate language that was intentionally conversational to reinforce a peer‐to‐peer learning experience as opposed to an expert‐driven experience. The consortium developed personas based on components of the ideation model (Figure 3). For example, formative research provided insight into caregivers’ cognitive perceptions and emotional response. The recipe book's personas communicated key messages in a nondirective way by sharing knowledge while acknowledging these perceptions and emotional responses. The personas use concepts from MI to craft supportive and empathetic messages, the nudge theory to provide cues for positive decision making, and the socioecological model by including a husband (relationship sphere) and a community health worker (community sphere).

The ideation model also informed the book's focus on the knowledge and skills needed to increase dietary diversity and support CF through breastfeeding and kitchen hygiene guidance. Knowles’ four adult learning principles informed key features of the book (Knowles, 1978):

1. Involvement: the book is organized so users can identify topics of most interest to them.
2. Experience: The book acknowledges, celebrates, and builds on caregivers’ knowledge and expertise.
3–4. Problem‐centred and immediately relevant: Formative research uncovered issues of immediate concern and the book used a problem‐centred approach to address them. For example, caregivers felt they had no need for a cookbook. According to interviews, caregivers desired guidance on what to cook rather than instruction on how to cook. Thus, the book was rebranded as a recipe book as opposed to a cookbook and the content focused on increasing dietary diversity and nutrient density rather than teaching caregivers to cook.

Adult learning theory also states that adults are internally motivated to learn. Thus, the book includes motivational messages to encourage a readiness and motivation to learn.

Throughout the book, the nudge theory was most obviously employed by using familiar, traditional recipes and modifying them to improve dietary diversity. This modification provides caregivers with guidance on how to improve IYCN in a familiar format, so changes feel incremental and achievable. The adaptation of familiar, traditional recipes also provided caregivers with the tools to increase dietary diversity, reduce micronutrient deficiencies and provide easily understandable ways to make healthy dietary substitutions based on availability, affordability, and personal preference.

### 3.4 Step 4: Purpose‐driven visual communication

The book's visual communication strategy was intended to achieve three things: (1) increase self‐efficacy, (2) improve accessibility and (3) ensure acceptability. Visual communication techniques include iconography‐style illustrations and simple, graphic images to increase a user's learning and memory (Bobek & Tversky, 2016).

**Figure 4:**
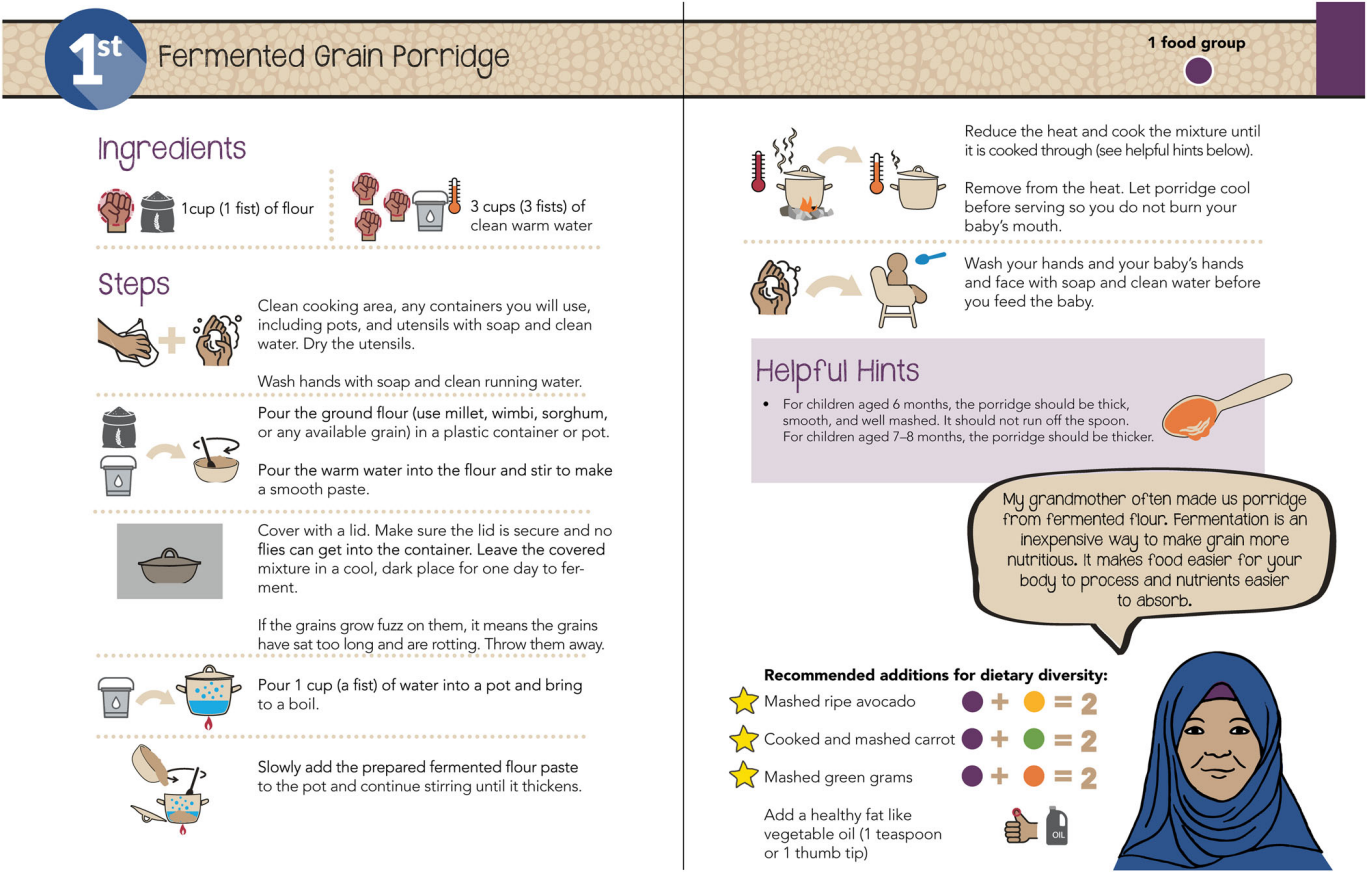
Illustrating key concepts in a recipe.

The book also utilizes binary illustrations that show users both what to do and what not to do. This approach clearly defines inappropriate actions and provides alternative appropriate actions. By focusing on replacing one action with another, the binary illustrations do not overwhelm the reader with choices, avoiding the paradox of choice, the theory that decision‐making gets more difficult as choices increase (Brockett, 2006). Figures 3 and 4 provide examples of this strategy used in the book's food safety and hygiene sections.

The book uses simple icons consistently to reinforce concepts. Consistency helps illustrate key concepts and reinforce important messages such as illustrating hand washing as the first and last step of every recipe (Figure 4). Icons are also used to help users quickly identify ingredients that support specific physiological systems and ingredients that are both affordable and nutrient dense (Figures 2 and 4).

Another important visual element is the book's strong colour palette and brand. The palette and brand were developed specifically for the priority population. The colour palette took its inspiration from the colours of the Kenyan savannah, Masai beading, kikoys and traditional baskets. This palette was intended to feel familiar and celebrate Kenyan identity (Park & Tang, 2019). The branding uses a wax print‐style motif used in Kenyan textiles.

The use of photography was purposefully limited because it is more difficult to clearly provide instruction through photography. Instead, the consortium used positive photographs to reinforce positive emotions, improve engagement and motivate the priority population (Rawolle et al., 2016). The photographs are of healthy infants and children in loving embraces with their mothers, fathers, or grandparents.

### 3.5 Step 5: Tailored to audience

The scoping desk review identified CF feeding gaps and opportunities that the consortium explored further during primary data collection. These gaps and opportunities were used when developing the content of the book. For example, the Comprehensive Nutrient Gap Assessment (CONGA) conducted as part of the RISING project found that CF‐aged children do not consume adequate iron, vitamin A, zinc or B12. After the CONGA was completed, an Affordability Study was conducted. The study identified affordable whole foods that would address common micronutrient gaps for children 6–23 months of age. The consortium ensured recipes and meal plans included these affordable ingredients. They are highlighted throughout the book using an icon. Other examples include suggested seasonal substitutions, the book's use of nontechnical language and familiar design motifs.

The book's design included graphics and photos representing all types of caregivers. The personas demonstrate this technique by including different genders, ages and religions. The consortium carefully crafted the personas and their messages to ensure they represented empowered individuals who were making positive behaviour change. The personas were careful not to challenge social norms too strongly, instead demonstrating positive changes within realistic and culturally appropriate scenarios.

## 4 DISCUSSION

The ADAPT approach facilitated the production of an evidence‐based, interdisciplinary, and innovative recipe book. Its key differentiation from other cookbooks is the comprehensive approach to CF as well as the purposeful use of visual communication to facilitate access, understanding, retention and application of key messages to elicit behaviour change.

As noted, previous cookbooks focused on recipes whereas this book focuses on practical guidance to support CF. It recognizes that for behaviour change to happen, users must understand, find value in and be willing to adopt new behaviours (Knowles, 1978). As such, the book presents the benefits of proper CF and describes information in a user‐friendly way. Its content is tailored to caregivers’ wants and needs, and its approach to visual communication helps ensure understanding.

### 4.1 Addressing unexpected findings

Formative applied research uncovered some unexpected findings. Notably, existing IYCN products utilized visual design as opposed to visual communication. Visual design focuses on aesthetics, whereas visual communication focuses on conveying information, and improving understanding and retention. Visual communication also recognizes that we communicate in many ways, the written word being only one. Based on the dual coding theory, the brain codes and stores appropriate visuals in both the linguistic and visual areas of the brain which can enhance understanding and memory, increasing the likelihood of retention (Paivio, 2008). Other gaps included tailoring to the target audience both in terms of language and lived experience, and message consistency within products. These gaps created an opportunity to develop a rich, evidence‐based approach that capitalised on behaviour change theories and visual communication.

### 4.2 Strengths and limitations of the project

Primary strengths of this project include multistakeholder involvement, the use of behaviour change theory and visual communication to inform design and the use of robust primary and secondary research to target need and increase cultural sensitivity. This approach is innovative and interdisciplinary as it uses BCC in new ways and pulls from multiple BCC theories as well as adult learning and visual communication research. Its use of visuals to increase understanding and retention of key concepts is based on brain mapping and cognitive research (Paivio, 2008).

Another strength is the book's holistic approach to CF. The book thoughtfully addresses multiple barriers to CF by providing guidance on breastfeeding; age‐appropriate feeding frequency, consistency, and amounts; and food safety and hygiene. It addresses affordability by highlighting the most affordable nutrient‐dense ingredients and seasonality by suggesting seasonal substitutions. It encourages dietary diversity while acknowledging social and cultural preferences and acknowledges common concerns shared by caregivers. Caregivers can find all this information in a centralised place making it easier for them to access and to follow.

The ADAPT approach was the product of an integrated team of nutrition experts, researchers, BCC experts and visual communications practitioners. The richness of the team's skills and expertise lent itself to a strategic, fit‐for‐purpose product. Including behaviour change and visual communications experts in projects like this can help ensure that technical information and research findings are transformed into tools that empower and influence positive change.

Potential limitations include the remote nature of data collection which may have influenced the level of information collected. COVID‐19 restricted movement worldwide, thus the project was unable to conduct in‐person data collection activities. However, the authors are confident that triangulation of the RISING data, desk review and IDIs generated robust findings. We acknowledge that we may have identified additional findings if in‐person data collection had been feasible.

### 4.3 Lessons learned

This project was conducted during the height of the COVID‐19 pandemic. As such, in‐person meetings could not take place. In person meetings foster collaboration and trust. They provide a participatory environment that may more readily foster innovation. The team worked to foster that environment through virtual platforms but felt in‐person meetings would have been more effective. As COVID‐19 travel restrictions ease, similar projects should return to leveraging in‐person, participatory approaches to product development.

### 4.4 Next steps and future research

The next step for this work is to promote and disseminate the recipe book. Interviewees shared that while there are existing IYCF‐related interventions across Kenya, a lack of human and financial resources in certain locations limits caregivers’ access to counselling and training. Marketing the recipe book as a take‐home behaviour change tool will encourage its use and promotion among health workers.

The consortium carefully considered affordability when developing the recipe book's strategy. Thus, the dissemination plan encourages marketing the financial benefits of the recipe book in addition to the health benefits. Respondents also recommended the use of influential opinion leaders to promote the book, and social platforms to disseminate the book. Promotion strategies should use the ADAPT approach to ensure they are evidence‐based, impact‐driven, and audience appropriate.

Promotion, use and perceptions of the recipe book must be evaluated after dissemination to determine the book's ability to affect long‐term behaviour change and nutritional status. Conducting a longitudinal study examining the potential impact of the recipe book would be helpful in assessing the recipe book's effectiveness. In addition to evaluation of the recipe book specifically, there is need for further research examining the relationship between visual communication and behaviour change broadly. Visual communication is used widely across sectors, but the evidence base is limited. Potential studies include those that explore (1) which types of visual communication strategies facilitate understanding, retention and adoption of new behaviours and (2) how design elements impact the acceptability of materials.

Visual communication for behaviour change is not yet a widely understood concept in public health. Most existing public health visual communication focuses on data visualization as opposed to behaviour change. Visual communication that does focus on behaviour change is often limited to communicating risk rather than building skills (CDC, 2022). There is a need for more training on and research into visual communication, and more visual communications experts incorporated into public health efforts. Finally, the ADAPT approach should be adapted to other contexts, evaluated and improved upon. For example, the team had hoped to conduct participatory action research. However, COVID‐related travel restrictions required the team to use applied research. The team would like to test the ADAPT approach using action research and welcomes the adoption, adaptation and evaluation of the approach by other public health practitioners.

## 5 CONCLUSION

ADAPT's first two steps (formative applied research and dialogue with stakeholders) ensured a context‐specific understanding of CF in Kenya. The consortium used behaviour change theory to ensure research findings were addressed using evidence‐based strategies. Visual communication translated educational messages and instruction into graphics that foster understanding and retention of key concepts. Finally, the content and visuals were tailored to ensure they were specific and appropriate to the audience. The novelty of this systematic and evidence‐based approach lies in its integration of BCC theory and visual communication best practice.

The ADAPT approach fostered the development of a strategic, rich and fit‐for‐purpose recipe book that thoughtfully and holistically addresses a wide range of barriers, providing practical solutions and increasing self‐efficacy around CF. The approach needs validation and further research but could provide a systematic way to integrate elements proven to foster positive change in both nutrition work and public health efforts more broadly.

## AUTHOR CONTRIBUTIONS

Alyssa Lowe, Ann DiGirolamo, Esther Omosa, Frida Okeyo, and Lawrence Odollo designed the research study. Esther Omosa, Frida Okeyo, and Lawrence Odollo collected the data. Alyssa Lowe, Amy Callis, Amma Boakye, and Esther Omosa analyzed the data. Alyssa Lowe, Amy Callis, and Ann DiGirolamo wrote the paper. Amy W. Girard, Esther Omosa, Frida Okeyo, Amma Boakye, Esther Omosa, Lawrence Odollo, Penjani Kamudoni, Patrick Codjia, Betty Samburu, Caroline Arimi, Wendy Gonzalez, and Laura Kiige reviewed the paper.

## CONFLICT OF INTEREST STATEMENT

The authors declare no conflict of interest.

## Supporting information

Supporting information.

## Data Availability

Data available on request from the authors.
