# Supplementary material for: Evidence‐based complementary feeding recipe book for Kenyan caregivers: A novel approach
Source: Matern Child Nutr. 2023 Oct 3;20(Suppl 3):e13475. doi: 10.1111/mcn.13475 (PMC10782138; doi:10.1111/mcn.13475)
Supplement: Supplementary file 1 — Supporting information. [file MCN-20-e13475-s001.docx]

Supplemental Material

Table 2: Counties and sub-counties where data were collected by region

| **Sub-County Selected for Cook-Book Data Collection** | | | |
| --- | --- | --- | --- |
| **Region** | **County selected** | **Sub-counties** | **Sub-counties selected** |
| Savannah | 1. Migori | Rongo, Awendo, Suna East, Uriri, Nyatike, Kuria West, Kuria East | Awendo |
|  |  |  | Uriri |
|  |  |  | Kuria |
|  | 2. Uasin Gishu | Turbo, Soy, Moiben, Ainabkoi, Kapsaret, Kesses | Soy |
|  |  |  | Moiben |
|  |  |  | Ainaboi |
|  |  |  | Kapsaret |
|  |  |  | Turbo |
|  | 3. Homa Bay | Kasipul Kabondo, Kabondo Kasipul, Karachuonyo, Rangwe, Homabay Town, Ndhiwa, Mbita, Suba | Kabondo Kasipul |
|  |  |  | Karachuonyo |
|  |  |  | Homabay town |
|  |  |  | Suba |
|  | 4. Kisii | Bonchari, South Mugirango, Bomachoge Chache, Nyaribari Masaba, Bomachoge Burabu, Bobasi, Nyaribari Chache, Kitutu Chache North, Kitutu Chache South. | South Mugirango, |
|  |  |  | Nyaribari Chache, |
|  |  |  | Kitutu Chache North |
|  |  |  | Nyaribari Masaba |
|  | 5. Nairobi | Westlands, Dagoretti North, Dagoretti South, Lang’ata, Kibra, Roysambu, Kasarani, Ruaraka, Embakasi South, Embakasi North, Embakasi Central, Embakasi East, Embakasi West, Madaraka, Kamkunji, Starehe, Mathare | Mathare |
|  |  |  | Kibra |
|  |  |  | Dagoretti South |
|  |  |  | Embakasi |
|  |  |  | Makadara |
|  | 6. Busia | Mount Elgon, Sirisia, Kabuchai, Bumala, Kanduyi, Webuye East, Webuye West, Kimilili | Mount Elgon |
|  |  |  | Bumala |
|  |  |  | Kanduyi |
|  |  |  | Webuye West |
|  | 7. Murangá | Kangema, Mathioya, Kiharu, Kigumo, Maragwa, Kandara, Gatanga | Kangema |
|  |  |  | Kigumo |
|  |  |  | Maragwa |
| Arid/Desert | 8. Wajir | Wajir North, Wajir East, Trabaj,Wajir West, Eldas, Wajir South | Eldas |
|  |  |  | Trabaj |
|  |  |  | Wajir North |
|  | 9.West pokot | Kapenguria, Sigor, Kacheliba, Pokot South | Kapenguria |
|  |  |  | Sigor |
|  | 10. Kajiado | Kajiado North, Kajiado Central, Kajiado East, Kajiado West, Kajiado South | Kajiado Central |
|  | 11. Baringo | Tiaty, Baringo North, Baringo Central, Baringo South, Mogoito, Eldama Ravine | Baringo North, Baringo South, |
|  | 12. Makueni | Mbooni, Kilome, Kaiti, Makueni, KibweziWest, Kibwezi East | Mbooni |
|  |  |  | Kibwezi West |
|  | 13. Tharaka Nithi | Chuka/Igambang’ombe, Tharaka | Chuka |
| Coast | 14. Mombasa | Changamwe, Jomvu, Kisauni, Nyali, Likoni, Mvita | Changamwe Nyali |
|  | 15. Taita Taveta | Taveta, Wundanyi, Mwatate, Voi | Taveta, Voi |
|  | 16. Lamu | Lamu East and Lamu west | Lamu East |
